# Influence of Ion Chelating Agents with Different Chelating Abilities on the Properties and Microstructure of Cement-Based Materials

**DOI:** 10.3390/ma18102256

**Published:** 2025-05-13

**Authors:** Ke Zhao, Ruiyang Wang, Jianying Yu, Quantao Liu, Yagang Zha

**Affiliations:** 1 School of Materials Science and Engineering, Chang’an University, Xi’an 710061, China; kzhao@chd.edu.cn; 2 State Key Laboratory of Silicate Materials for Architectures, Wuhan University of Technology, Wuhan 430070, China; jyyu@whut.edu.cn (J.Y.); liuqt@whut.edu.cn (Q.L.); zhayagang1023@163.com (Y.Z.)

**Keywords:** ion-chelating agent, chelation ability, microstructure of cement-based materials, impermeability

## Abstract

Concrete structures are prone to cracking and seepage issues due to material degradation during long-term service. Ionic chelating agents (ICAs) can significantly enhance the durability and extend the service life of concrete structures by chelating metal ions in the cement matrix and promoting the formation of crystalline products within pores. The study selected commonly used ICAs, including sodium gluconate, sodium maleate, and sodium citrate, as well as a self-made high-efficiency ICA, to compare their chelating abilities for metal ions (such as Al^3+^, Mg^2+^, Fe^3+^, and Ca^2+^). Their effects on the performance and microstructure of cement-based materials were evaluated through tests on hydration heat, mechanical strength, the chloride ion diffusion coefficient, pore size distribution, and microstructural analysis. The results showed that the stronger the chelating ability of the ICA, the more significant its improvement on the performance and microstructure of cement-based materials. Cement paste incorporating the high-efficiency ICA exhibited significantly accelerated hydration kinetics, with the hydration rate markedly increasing and the peak heat release rising from 0.0012 W/g to 0.0016 W/g, thereby effectively enhancing the early-age properties of the cement-based materials. After 28 days, compared to ordinary mortar, the flexural and compressive strengths of mortar containing the high-efficiency ICA increased by 17.1% and 11.6%, respectively, while the chloride ion diffusion coefficient decreased by 37.4%. Pore size distribution and microstructural analyses indicated that mortar incorporating the high-efficiency ICA exhibited the most compact internal structure, with abundant crystalline products such as CaSiO_3_ and 3CaO·Al_2_O_3_·3CaSO_4_·32H_2_O (AFt) forming within the pores. These findings suggest that optimizing the ion-chelating capacity of ICA provides a feasible strategy to enhance the compactness, durability, and mechanical performance of cement-based materials in practical engineering applications.

## 1. Introduction

Concrete is the most commonly used building material in architecture, and its significance cannot be overlooked [1]. As a fundamental construction material, concrete finds extensive application in diverse infrastructure projects, including bridges, roadways, tunneling systems, and skyscrapers, owing to its exceptional mechanical properties, long-term durability, and economic advantages [2]. However, this indispensable material undergoes progressive degradation throughout its service life, particularly when exposed to fluctuating environmental conditions. Such deterioration, with water penetration being a primary manifestation, substantially threatens both structural integrity and operational performance [3]. Concrete permeability, defined as the migration of liquids through microscopic voids, fractures, or structural interfaces, serves as a critical indicator of compromised durability. This phenomenon not only reduces load-bearing capacity but also impairs architectural functionality and visual appeal while simultaneously contributing to internal material breakdown [4]. This phenomenon not only weakens the mechanical strength of concrete but also accelerates the corrosion of internal reinforcement, ultimately leading to structural failure.

The primary cause of concrete leakage is its high porosity, as internal capillary pores and microcracks provide pathways for moisture infiltration. Under external water pressure or fluctuating humidity, this can easily lead to structural damage [5]. Environmental and mechanical factors, particularly structural loads, temperature variations, and cyclic freeze–thaw processes, significantly contribute to crack formation and propagation. These defects create penetration channels that allow water and corrosive ions to infiltrate deeper into the concrete matrix [6]. Furthermore, chemicals like chlorides and sulfates penetrate the concrete and react with the matrix, producing expansive or corrosive products that further exacerbate damage and leakage [7]. To address the issue of concrete leakage, current solutions primarily focus on enhancing the density of concrete, improving its pore structure, and increasing its crack resistance. Common measures for improving concrete impermeability include adjusting the ratios of cement, water, and aggregates, thus improving concrete density; adding admixtures such as expanding agents, water reducers, and air-entraining agents to reduce porosity; and applying waterproof coatings or sealants to the surface of concrete to prevent the penetration of moisture and chemicals, extending the service life of the concrete [8,9,10]. However, these methods have limitations. For instance, lowering the water–cement ratio may affect workability, the effectiveness of admixtures can be inconsistent under varying environmental conditions, and surface coatings may age or fail, making them insufficient for long-term leakage prevention.

**Figure 1:**
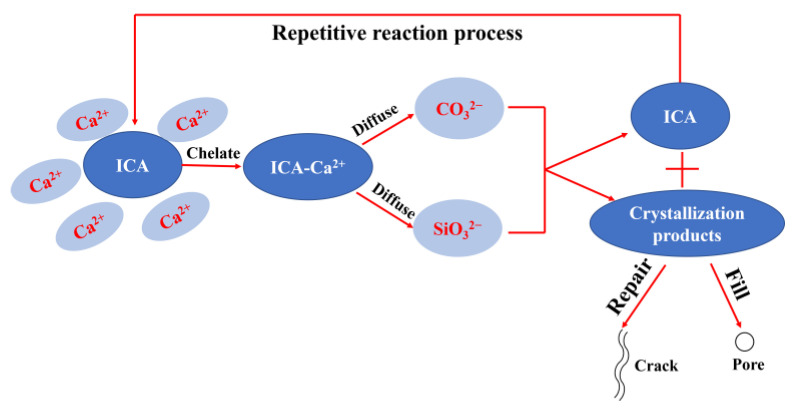
ICA reaction process in concrete [17].

As concrete waterproofing technology evolves, permeable crystalline waterproofing admixtures have emerged as a widely used and effective waterproofing method [11,12]. The crystalline permeable waterproofing admixture enhances the impermeability of concrete through hydrophilic chemical activation. When its active components (primarily silicate compounds) react chemically with calcium hydroxide and moisture in the cement matrix, they generate insoluble crystalline substances—mainly calcium silicate hydrate. These crystals physically block capillary pores and micro-cracks, forming a permanent hydrophobic barrier through crystal growth. Upon water penetration, the self-healing function is activated, thereby improving the impermeability and durability of cement-based materials [13,14]. Penetrative crystalline admixtures are categorized into two types based on their waterproofing methods. One is Na_2_SiO_3_, Ca(HCO_3_)_2_, and active SiO_2_ as the main components of the crystalline admixture. This type of admixture reacts with cement hydration products to produce CaSiO_3_ and CaCO_3_ crystals that fill pores and repair cracks, enhancing the density and impermeability of concrete. However, these admixtures are gradually consumed during the reaction process, and their effectiveness diminishes over the service time of the concrete [15,16]. The second type is a crystallization admixture that contains organic matter with the ability to chelate metal ions as the main ingredient. This type of admixture can chelate free Ca^2+^ in concrete to form a more stable chelate and migrate within the concrete to react with the hydration products in the pores of the concrete to generate CaSiO_3_ and CaCO_3_ crystals that continue to build up and ultimately form a dense network structure. As the crystalline products grow, the pores in the concrete are gradually filled, limiting moisture paths and increasing the strength and impermeability of the concrete. Unlike the first category, the ion-chelating agent (ICA) is not consumed during the process, providing long-term effectiveness in improving concrete performance [17,18]. The reaction process of the ICA within concrete is shown in Figure 1.

Due to their long-lasting effects and overall waterproofing performance, ICAs are considered to have significant advantages among crystalline additives [19]. At present, domestic and international research on ICAs mainly focuses on their effects on the self-repair of cracks in cement-based materials, internal microstructure changes, mechanical strength, and durability. Investigations have shown that these admixtures effectively enhance crack self-repair, waterproofing, and durability in cement-based materials [20,21,22]. However, the different types of chelation-based crystalline admixtures, each with its own ICA as the core component, affect the performance and microstructure of cement-based materials differently. According to the crystallization mechanism of ICA, variations in the number of functional groups result in differences in the capacity and rate of chelating metal ions (e.g., Ca^2+^, Mg^2+^, and Al^3+^) in cement-based materials. These differences influence the effectiveness of the precipitation reaction in filling pores and repairing microcracks. However, existing ICAs used to improve the impermeability and self-repairing capabilities of cement-based materials still face challenges, such as limited ion-chelating capacity, slow migration rates, and difficulties in quickly filling pores and repairing microcracks [23]. Building on previous research, our laboratory synthesized a high-efficiency ICA using maleic anhydride as a precursor, designed through molecular structure optimization [24]. This ICA can significantly promote the development of crystallization precipitation at the cracks and effectively enhance the self-repairing capability of cement-based materials, but the chelation ability of this high-efficiency ICA for different metal ions has not been thoroughly investigated [25]. Therefore, it is necessary to compare the differences in the metal ion-chelating ability of high-efficiency ICA and existing ICAs and, based on this, comprehensively analyze the effect of different ICAs on the performance and microstructure of cement-based materials in order to explore the interrelationship between the chelation ability of ICAs and the enhancement of the structure and performance of cement-based materials, which is critical for extending the service life of these materials.

In this paper, ICAs containing different numbers of chelation sites—sodium gluconate (A1), sodium maleate (A2), and sodium citrate (A3)—were selected, and the chelating capacity of these ICAs and a high-efficiency ICA (An) for Ca^2+^ was tested. The ion-chelation ability of the ICAs was evaluated through the study of the conductivity reduction rate of the solutions containing ICAs at different temperatures, pH, and ion conditions. Additionally, the influences of different ICAs on the performance and microstructure of cement mortar were researched through parameters such as heat of hydration, flexural and compressive strength, chloride ion diffusion coefficient, pore size distribution, and internal microstructural morphology.

## 2. Materials and Methods

### 2.1. Materials

**Figure 2:**
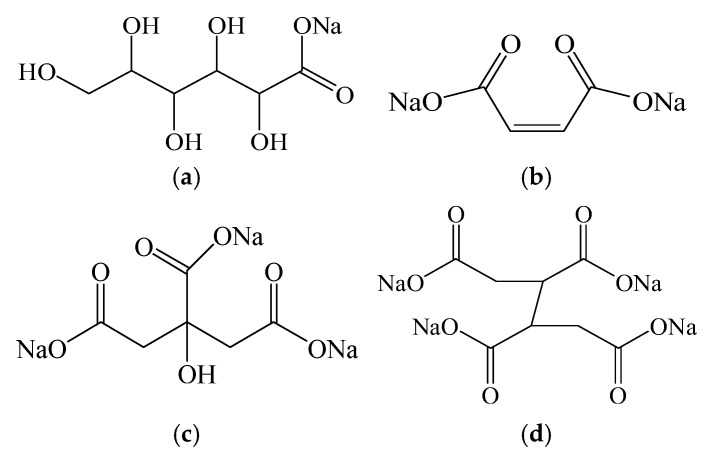
Molecular structures of different ICAs: (**a**) A1; (**b**) A2; (**c**) A3; and (**d**) An.

The cement was PO42.5 ordinary Portland cement, produced by Conch Cement Co., Ltd. (Xianyang, China). Natural river sand with a fineness modulus of 2.35 was supplied by Xi’an Gaoke Building Materials Technology Co., Ltd. (Xi’an, China). The selected ICAs were A1, A2, and A3, obtained from Sinopharm Chemical Reagent Co., Ltd. (Shanghai, China). The high-efficiency ICA (An) is a white powder synthesized from maleic anhydride, hydrogen peroxide, and sodium hydroxide [24]. Additional experimental materials included maleic anhydride, calcium hydroxide, hydrogen peroxide, magnesium chloride hexahydrate, sodium hydroxide, ferric chloride hexahydrate, aluminum hydroxide, disodium ethylenediaminetetraacetate (EDTA), ammonium chloride, silver nitrate, anhydrous calcium chloride, and ammonia solution, all of which were analytical-grade reagents procured from Sinopharm Chemical Reagent Co., Ltd. Hydrochloric acid was sourced from Xinyang Chemical Reagent Plant (Xinyang, China), and Eriochrome black T indicator was supplied by Jining Tainuo Chemical Co., Ltd. (Jining, China). The chemical composition of the raw cement is presented in Table 1, and the molecular structures of the different types of ICAs are shown in Figure 2.

### 2.2. Chelating Capacity Test

#### 2.2.1. Preparation of Test Solutions

Various ionic solutions were prepared for testing the chelating ability of ICAs on metal ions.

(1) Ca(OH)_2_ (1.0 g) was dissolved in 100.0 mL of deionized water, stirred vigorously, and allowed to fully dissolve and then stand. Vacuum filtration was performed to remove undissolved solids and obtain a saturated Ca(OH)_2_ solution. The pH of the saturated Ca(OH)_2_ solution was adjusted to 11, 9, and 7 by slowly adding HCl to prepare Ca(OH)_2_ solutions with different pH levels for studying the chelating ability of ICAs on different Ca^2+^ ion concentrations.
(2) Solutions of Al^3+^, Mg^2+^, Fe^3+^, and Ca^2+^ ions were prepared using Al(OH)_3_, MgCl_2_·6H_2_O, FeCl_3_·6H_2_O, and CaCl_2_, respectively.
(3) Next, 0.1 mol/L solutions of A1, A2, A3, and An were prepared, and 10.0 mL of the ICA solution was mixed with 10.0 mL of other related metal ion solutions to prepare different ICA–metal ion mixed solutions.
(4) A quantity of 9.25 g of EDTA was accurately weighed and placed in a 500 mL beaker, and 500 mL of distilled water was added to make a 0.05 mol/L solution. Next, 5.4 g of NH_4_Cl was weighed and dissolved in water; 36 mL of NH_3_·H_2_O was added, and the solution was diluted to 100 mL to obtain an ammonia–ammonium chloride buffer solution for testing Ca^2+^-chelating capacity.

#### 2.2.2. Calcium Ion-Chelating Capacity Test

Ion-chelation capacity is a useful visual measure for determining the ability of an admixture to bind ions. The larger the ion-chelating capacity, the stronger the chelating ability of the admixture for ions, so the ion-chelating capacity of the admixture was tested to compare the ion-carrying capacity of different ICAs. To conduct this test, 0.1 g of the ICA was accurately weighed and dissolved in a small amount of distilled water, and 10 mL of a 0.1 mol/L CaCl_2_ solution was added. The mixture was shaken thoroughly, and then 10 mL of ammonia–ammonium chloride buffer solution and 4–5 drops of Eriochrome black T indicator were added. This was titrated with 0.05 mol/L EDTA standard solution until the solution turned pure blue, with the reaction process lasting approximately 3 min. The Ca^2+^ chelation capacity (*L*) is calculated as follows:

(1) $$L=\frac{40\times (10c_{1}-c_{2}v)}{m}$$

where *c*_1_ is the Ca^2+^ solution’s concentration (mol/L), *c*_2_ is the EDTA solution’s concentration (mol/L), *v* is the EDTA solution’s volume (mL), *m* is the ICA’s mass (g), and *L* is the ICA’s Ca^2+^ chelation capacity (mg/g).

#### 2.2.3. Test of Chelating Ability of Ion-Chelating Agents for Different Cations

The ICA has a certain chelating ability to the cations in the solution due to the structure of the functional groups in its molecule, resulting in a decrease in the number of ions in the solution and, consequently, a decrease in the conductivity of the solution. Therefore, the chelating ability of the ICA for ions can be evaluated by observing the conductivity decline process of the solution over time. The electrodes were cleaned with deionized water before conducting the conductivity test. The cleaned electrode was placed in a mixture with an ICA, and the values were recorded when the readings stabilized. The conductivity test was carried out at one-hour intervals. The decrease in solution conductivity was calculated as follows:

(2) $$E_{c}=\frac{E_{cn}-E_{c0}}{E_{c0}}\times 100\%$$

where *E_c_* is the percentage reduction in conductivity (%), *E_cn_* is the solution conductivity after *n* h (μs/cm), and *E_c_*_0_ is the initial conductivity of the solution (μs/cm).

#### 2.2.4. Saturated Calcium Hydroxide Turbidity Point Test

The chelation of calcium ions by ICA disrupts the dissolving equilibrium of Ca(OH)_2_ in water, increasing its solubility. Because the solubility of Ca(OH)_2_ is inversely proportional to temperature, the chelation stability of ICA on Ca^2+^ can be evaluated by performing the turbidity point test of saturated Ca(OH)_2_ solutions containing ICA. To conduct the test, 0.1 mol of the ICA was added to 100.0 mL of a saturated, clear Ca(OH)_2_ solution and stirred until fully dissolved. Then, the Ca(OH)_2_ solution mixed with the ICA was heated until the solution became turbid, and the temperature at which turbidity appeared was recorded.

### 2.3. Preparation and Curing of Cement Mortar

**Figure 3:**
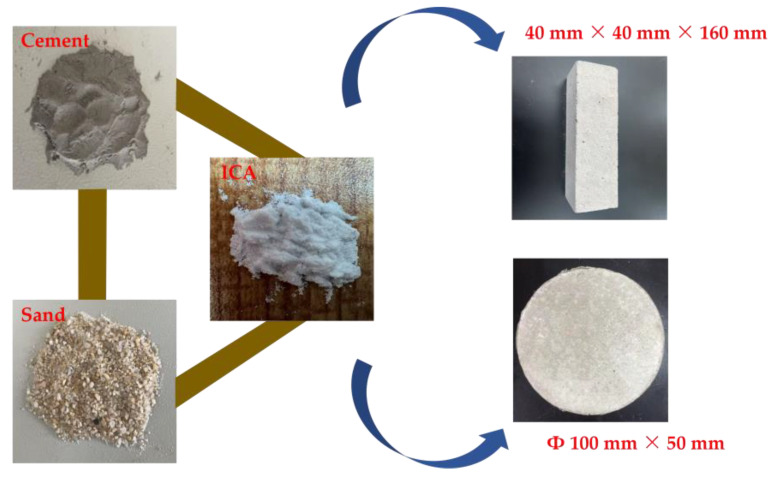
Specimen preparation procedure.

In this experiment, mortar was made with a water-to-cement ratio of 0.4 and a cement-to-sand ratio of 2. Based on preliminary exploratory experiments and literature references, the ICA dosage was selected as 0.10% of the cement mass [26]. The mortar mix proportions are shown in Table 2. During preparation, cement and sand were first added to the mixer and stirred for approximately 20 s. The ICA, dissolved in water, was then added, followed by mixing for 180 s. The mixture was cast into molds with dimensions of 40 mm × 40 mm × 160 mm and Φ 100 mm × 50 mm. After 24 h, the mortar was demolded and cured in a standard curing room. The specific sample preparation procedure is shown in Figure 3.

### 2.4. Performance Testing and Characterization

This section systematically investigates the influence of varying ICA dosages (0–0.1%) on cement mortar properties through mix proportion design methodology. The hydration heat evolution of cement paste over 75 h was measured using TAM-Air microcalorimetry. Mechanical property tests were conducted to evaluate the development of flexural and compressive strengths at 3, 7, 14, and 28 days of curing. The Rapid Chloride Migration (RCM) method was employed to determine chloride ion diffusion coefficients, characterizing the specimens’ impermeability. Nuclear magnetic resonance (NMR) technology was utilized to analyze pore structure characteristics in 28-day cured mortar. Furthermore, SEM-EDS microanalysis was applied to elucidate ICA’s effects on hydration product morphology and pore composition.

#### 2.4.1. Heat of Hydration Test

**Figure 4:**
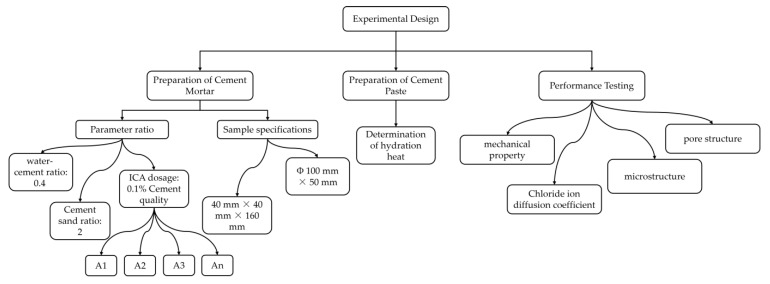
Experimental framework for cement specimen study.

A TAM-Air eight-channel microcalorimeter was used to assess the cement paste’s heat release rate and total heat release over a 75-h hydration period. The test employed cement paste with a water-cement ratio of 0.4, and the ICA dosage was fixed at 0.1% of the total cement mass. The cement pastes were identified as C1, C1-A1, C1-A2, C1-A3, and C1-An. The testing temperature was kept at 20 ± 2 °C. The experimental workflow framework is presented in Figure 4.

#### 2.4.2. Mechanical Properties Test

In this test, the mechanical properties of mortar were evaluated with reference to standard GB/T17671-2021 [27]. A fully automatic pressure testing machine was used to measure the flexural and compressive strengths of mortars containing different types of ICAs at 3, 7, 14, and 28 d, respectively. The speeds of the flexural and compressive strength tests were 0.1 kN/s and 2.4 kN/s.

#### 2.4.3. Chloride Ion Diffusion Coefficient Test

The chloride ion diffusion coefficient (CIDC) of mortar was determined using the rapid chloride migration coefficient method (RCM) in GB/T 50082-2009 [28]. A cylindrical specimen measuring Φ100 mm × 50 mm underwent vacuum water saturation before being tested on a concrete CIDC tester. At the final stage of the test, the specimens were split, and a 0.1 mol/L AgNO_3_ solution was sprayed onto the fractures. After a time of rest, the specimen’s chloride ion diffusion depth was determined. The CIDC of the mortar can be calculated using Equation (3):

(3) $$D_{RCM}=\frac{0.239\times \left(273+T\right)L}{\left(U-2\right)t}(X_{d}-0.0238\sqrt{\frac{\left(273+T\right)LX_{d}}{U-2}})$$

where *D_RCM_* is the CIDC (×10^−12^ m^2^/s), *U* is the absolute voltage (V), *T* is the mean temperature of the anode solution (°C), *L* is the sample height (cm), *X_d_* is the average chloride ion diffusion depth (cm), and *t* is the test duration (h).

#### 2.4.4. Pore Structure Test

The pore structure development of 28-day cured cement mortar was quantitatively evaluated using a VTMR20–010V low-field nuclear magnetic resonance (NMR) analyzer (Suzhou Niumag Analytical Instrument Corp., Suzhou, China) [29]. The specific procedure was as follows: a 1 cm^3^ specimen was fully dried in an oven and then saturated using a vacuum water saturator. Finally, the NMR *T*_2_ spectra of the saturated specimen were measured, and the water’s relaxing duration reflected the size of the pores. Pore size distribution was calculated using Equation (4):

(4) $$\frac{1}{T_{2}}=\rho (\frac{S}{V}{)}_{pore}$$

where *T*_2_ is the water relaxation time in the pore space (s), (*S/V*)*_pore_* is the ratio of pore surface area to volume, and *ρ* is the surface relaxation rate (70 μm/ms).

#### 2.4.5. Microscopic Morphology Analysis

A ZEISS Gemini 300 SEM (ZEISS, Oberkochen, Germany) was used to observe the internal microstructure of the mortar. A specimen approximately 5 mm^3^ in size was prepared for testing after drying, and it was sputter-coated with Pt to provide a conductive surface for imaging. An EDS was used to characterize the composition of the products in the specimen pores, analyzing their elemental composition.

## 3. Results and Discussion

### 3.1. Chelating Ability Test of Ion-Chelating Agents

#### 3.1.1. Calcium Chelating Capacity of Different Types of Ion-Chelating Agents

**Figure 5:**
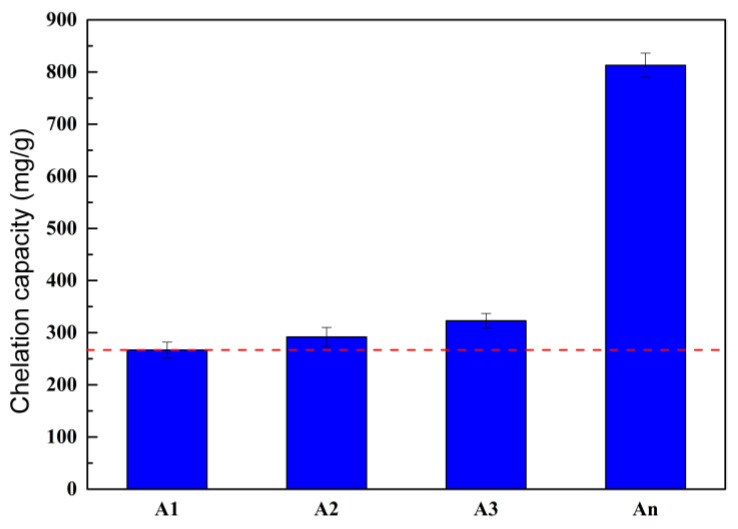
Ca^2+^-chelating capacity of different types of ICAs.

The Ca^2+^-chelating capacity of several ICA types is displayed in Figure 5. As shown in Figure 5, the Ca^2+^-chelating capacity gradually increases with the number of functional groups in the ICA’s molecular structure. The Ca^2+^-chelating capacities of A1, A2, and A3 were 267 mg/g, 292 mg/g, and 323 mg/g, respectively, while the Ca^2+^-chelating capacity of An reached up to 813 mg/g, showing an increase of 546 mg/g, 521 mg/g, and 490 mg/g compared to A1, A2, and A3, respectively. This indicates that, compared with the existing ICAs, the Ca^2+^-chelating capacity of An increased significantly, thereby effectively enhancing the ICA’s chelation crystallization effect.

#### 3.1.2. Calcium Ion Chelation Rates of Different Types of Ion-Chelating Agents

**Figure 6:**
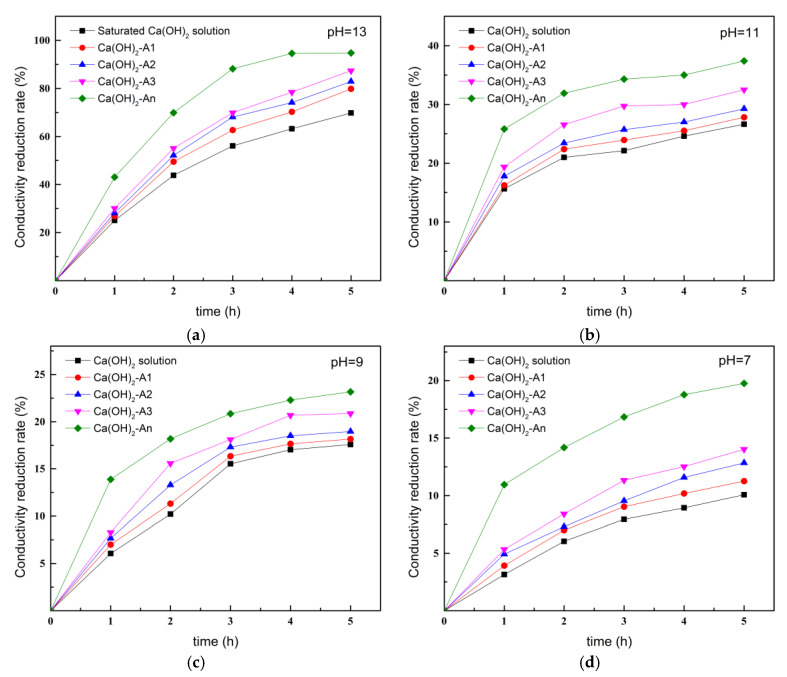
Conductivity variation of Ca(OH)_2_ solutions with ICAs at varying pH values: (**a**) pH = 13; (**b**) pH = 11; (**c**) pH = 9; and (**d**) pH = 7.

Figure 6 shows the trend of conductivity over time for Ca(OH)_2_ solutions containing ICAs at different pH values at normal temperature (20 °C). As shown in Figure 6a, the saturated Ca(OH)_2_ solution reacted with CO_2_ as it was left exposed to air for a prolonged period, resulting in turbidity. In the first 3 h, due to the higher concentration of Ca(OH)_2_ in the solution, the rate of conductivity decrease was initially faster; however, this rate gradually slowed in the later stages as reactive substances were depleted. At 5 h, the conductivity of the saturated Ca(OH)_2_ solution had decreased by 69.8%. In contrast, the decreases in conductivity of saturated Ca(OH)_2_ solutions containing ICAs were greater than that of the saturated Ca(OH)_2_ solution alone, with conductivities of Ca(OH)_2_-A1, Ca(OH)_2_-A2, Ca(OH)_2_-A3, and Ca(OH)_2_-An solutions decreasing by 87.4%, 82.9%, 79.8%, and 94.8%, respectively, at 5 h. This was primarily attributed to the different molecular structures of the ICAs; A1, A2, A3, and An each contained varying numbers of ion-chelating sites, with An having the highest number of chelating sites for Ca^2+^, demonstrating superior Ca^2+^-chelating ability.

Figure 6b–d illustrate the Ca^2+^-chelating ability of ICAs at pH levels of 11, 9, and 7. The decrease in conductivity of Ca(OH)_2_ solutions containing ICAs became less pronounced as the pH decreased due to the increased solubility of Ca(OH)_2_ in acidic conditions and the reduced solubility of CO_2_ from the air in the solution, resulting in a slower reaction rate. At pH 11, the conductivity of the Ca(OH)_2_ solution alone decreased to 26.7% after 5 h. Although the conductivity decreases of Ca(OH)_2_-A1, Ca(OH)_2_-A2, Ca(OH)_2_-A3, and Ca(OH)_2_-An solutions were also only 27.8%, 29.3%, 32.6%, and 37.4%, respectively, they still demonstrated a notable improvement compared to the Ca(OH)_2_ solution alone. At pH levels of 9 and 7, the Ca^2+^-chelating ability of ICAs weakened further, but the chelating ability of An remained superior to the other ICAs, which indicates that An exhibits the best chelating ability and speed for Ca^2+^ in different pH environments.

#### 3.1.3. Calcium-Chelating Ability of Ion-Chelating Agents at Different Temperatures

**Figure 7:**
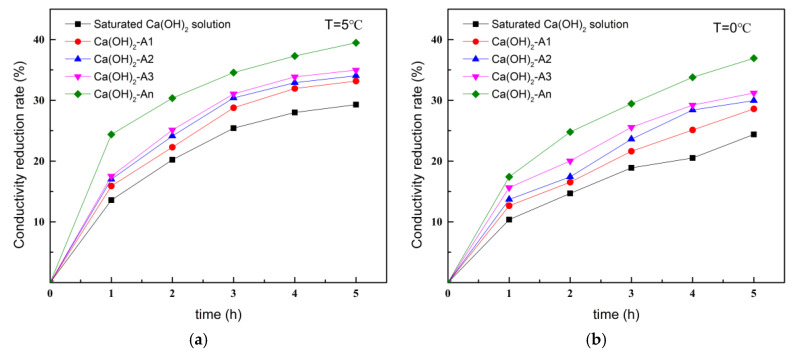
Conductivity changes in Ca(OH)_2_ solutions with different ICAs at various temperatures: (**a**) T = 5 °C; (**b**) T = 0 °C.

Figure 7 illustrates the variation curves of conductivity of saturated Ca(OH)_2_ solutions containing ICAs at temperatures of 5 °C and 0 °C. As the temperature decreased, the conductivity of the Ca(OH)_2_ solutions with dissolved ICAs also exhibited a declining trend, primarily due to the increased solubility of Ca(OH)_2_ at low temperatures, as well as slower molecular movement resulting in a lower rate of chelation reaction. Compared with Figure 7a, at a temperature of 20 °C, the decrease in conductivity of the mixed solutions of ICAs and Ca(OH)_2_ in air for 5 h was above 60%. Whereas, when the temperature was decreased to 5 °C, the decrease in conductivity of the mixed solutions in air for 5 h was less than 40%, where the conductivity of Ca(OH)_2_-A1, Ca(OH)_2_-A2, Ca(OH)_2_-A3, and Ca(OH)_2_-An solutions decreased to 33.2%, 34.1%, 34.9%, and 39.4%, respectively. The conductivities of the mixed solutions further decreased when the solution temperature was 0 °C. The conductivities of the Ca(OH)_2_-A1, Ca(OH)_2_-A2, Ca(OH)_2_-A3, and Ca(OH)_2_-An solutions decreased to 28.6%, 30.0%, 31.3%, and 37.0%, respectively, but were still significantly higher than those of the saturated Ca(OH)_2_ solution alone. This indicates that the ICAs maintained their Ca^2+^-chelating function at different temperatures, with An demonstrating the strongest chelating ability and rate, resulting in the fastest conductivity decrease in the Ca(OH)_2_-An solution.

#### 3.1.4. Chelating Ability of Ion-Chelating Agents for Different Metal Ions

**Figure 8:**
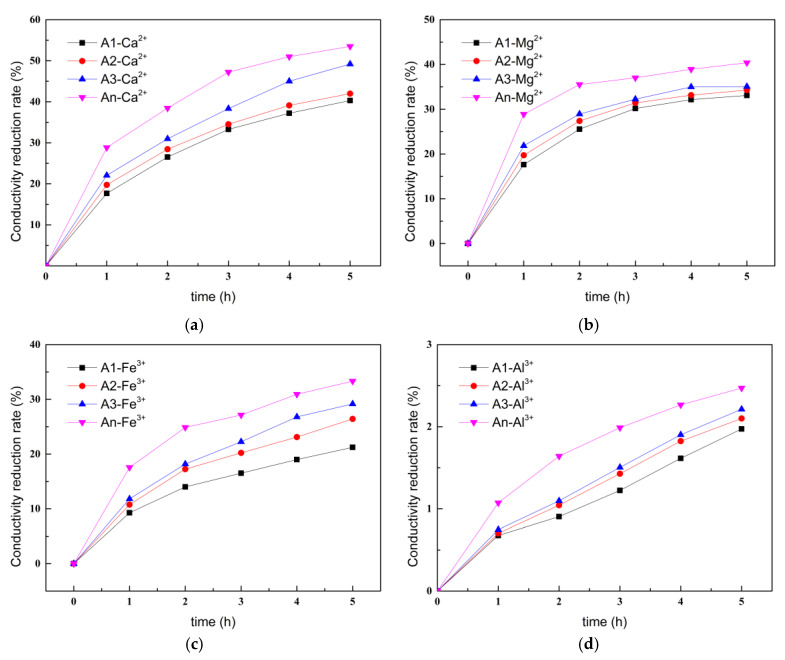
Variation of conductivity of different metal ion solutions containing ICAs: (**a**) Ca^2+^; (**b**) Mg^2+^; (**c**) Fe^3+^; and (**d**) Al^3+^.

Figure 8 shows the conductivity variation curve over time for metal ion solutions containing ICAs. As shown in Figure 6, the ICAs demonstrated stronger chelating ability for Ca^2+^, with conductivity reduction rates in calcium ion solutions mixed with A1, A2, A3, and An reaching 40.2%, 41.8%, 49.1%, and 53.4% after 5 h, respectively. The ICAs’ chelating ability for Mg^2+^ was somewhat weaker, with conductivity reductions in magnesium ion solutions mixed with A1, A2, A3, and An at 33.2%, 34.2%, 35.1%, and 39.0%, respectively, after 5 h. The conductivity reduction rates of the iron ion solution and aluminum ion solution dissolved with ICAs were significantly lower; specifically, the conductivity reduction rate of the aluminum ion solution mixed with ICAs was less than 3.0% after 5 h. This result was mostly explained by the lesser hydrolysis tendency and lower charge density of Ca^2+^ and Mg^2+^, allowing ICAs more opportunities to form stable chelates with them, whereas the high charge density and hydrolysis tendency of Fe^3+^ and Al^3+^ reduce their binding with chelators. Furthermore, among the ICAs, An exhibited the strongest chelating ability for Ca^2+^, Mg^2+^, Fe^3+^, and Al^3+^, due to the multiple chelating sites in its molecular structure.

#### 3.1.5. Turbidity Point of Saturated Calcium Hydroxide Solutions Containing Ion-Chelating Agents

**Figure 9:**
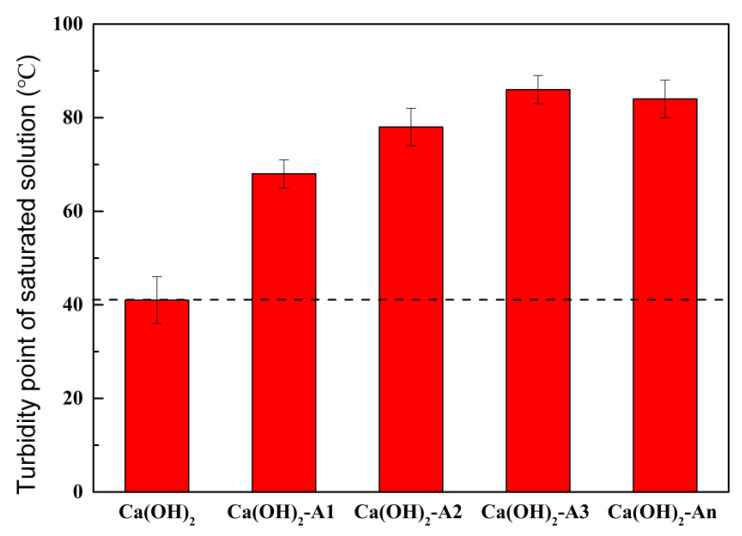
Turbidity points of saturated Ca(OH)_2_ solutions containing different ICAs.

Figure 9 shows the turbidity point of saturated Ca(OH)_2_ solution mixed with ICAs. The turbidity point of the solution substantially rose with the addition of ICAs. When the clarified saturated Ca(OH)_2_ solution was heated to 41 °C, turbidity appeared in the solution. The turbidity points of the clarified saturated Ca(OH)_2_ solutions containing 0.1 mol of A1, A2, A3, and An were 68 °C, 78 °C, 86 °C, and 84 °C, respectively. The saturated Ca(OH)_2_ solution containing A3 exhibited the highest turbidity point, which was mainly attributed to the strong stability and bond energy of the chelation formed between A3 and Ca^2+^. In contrast, the chelation formed between An and Ca^2+^ demonstrated good ion release capability.

### 3.2. Effect of Ion-Chelating Agents on the Properties and Microstructure of Cement Mortars

#### 3.2.1. Hydration Heat

**Figure 10:**
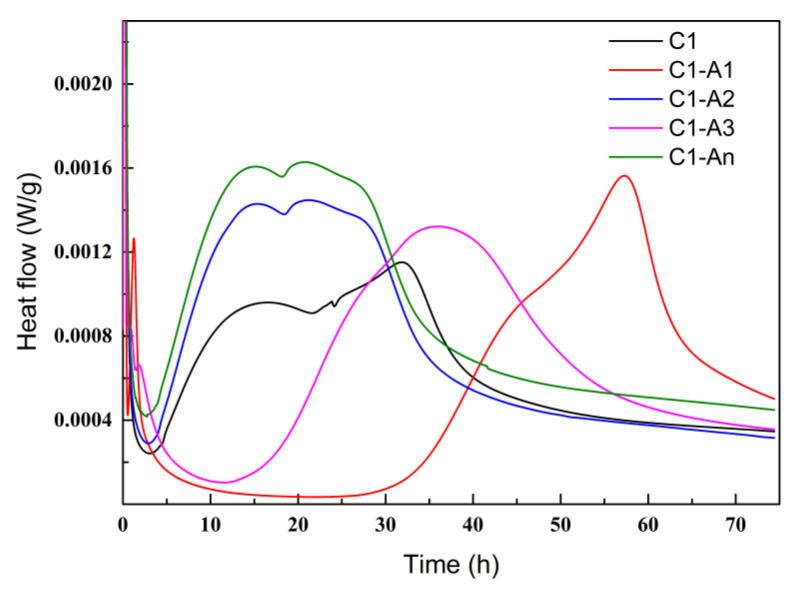
Cement paste hydration rate with various ICAs.

Figure 10 illustrates the hydration heat release rates of cement pastes with different ICAs. As shown, the heat release rate of C1 rose rapidly at the initial stage, reaching a peak before gradually decreasing. This characteristic peak intensity principally derives from the concurrent hydration processes of C_3_S and C_2_S [30,31]. When ICA was added, the cement hydration process exhibited distinct changes. For C1-A1, the heat release peak was notably delayed, indicating that A1 significantly slowed the rate of the cement hydration reaction. This retarding effect was primarily attributed to the hydroxyl and carboxyl groups in A1, which chelated Ca^2+^ in the early hydration stage and adsorbed onto cement particles, creating a barrier that delayed the hydration process. The heat release rate of C1-A2 was noticeably higher than that of C1, yet its curve shape remained similar, suggesting that A2 could chelate Ca^2+^ ions within the cement matrix, accelerating C_3_S decomposition and promoting the formation of hydration products, thereby increasing the heat release rate. The heat release peak of C1-A3 was delayed relative to C1, likely due to the greater stability of the chelate formed between A3 and Ca^2+^, which slowed the release of Ca^2+^ and exerted a retarding effect on cement hydration. For C1-An, the heat release rate was faster and exhibited a higher peak than C1-A2, which could be attributed to An’s weaker adsorption onto cement particles, preventing a strong barrier layer from forming. Additionally, an effectively chelated Ca^2+^ and released them promptly during reactions with cement components, facilitating the production of C-S-H and other products and thus accelerating the cement hydration process.

**Figure 11:**
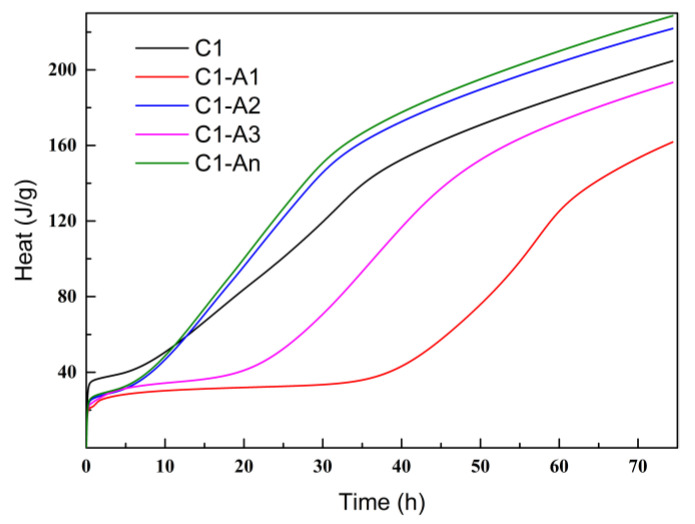
Cement pastes’ total heat with various ICAs.

The overall heat release of cement pastes with various ICAs is displayed in Figure 11. After 75 h of testing, the cumulative hydration heat of C1 was 204.7 J/g, while the cumulative heats of C1-A1, C1-A2, C1-A3, and C1-An were 161.8 J/g, 221.8 J/g, 193.3 J/g, and 228.6 J/g, respectively. Based on the heat release rates in Figure 8, it can be concluded that A1 and A3 formed relatively stable chelates with Ca^2+^, which slowed the heat release of the cement paste. In contrast, A2 and An released Ca^2+^ effectively upon chelation, allowing them to react with cement components and enhance hydration, thereby increasing the total heat release of the cement paste.

#### 3.2.2. Mechanical Strength

**Figure 12:**
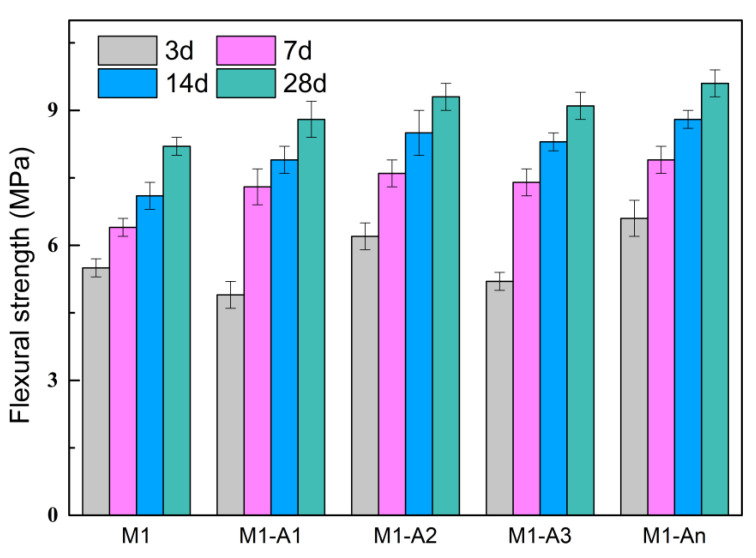
Flexural strength of mortars mixed with different ICAs.

Figure 12 presents the flexural strength of mortars with different ICAs at various curing ages. At 3 d, the flexural strength of M1 was 5.5 MPa, while the flexural strengths of M1-A1, M1-A2, M1-A3, and M1-An were 4.9 MPa, 6.2 MPa, 5.2 MPa, and 6.6 MPa, respectively. This difference can be attributed to the strong retarding effects of A1 and A3, which reduced the early flexural strength of the mortars relative to the ordinary mortar. In contrast, A2 and An had minimal retarding effects on cement and promoted the generation of early hydration products, further enhancing the early flexural strength of the mortars. After 28 d of curing, the flexural strength of M1 reached 8.2 MPa, while the flexural strengths of M1-A1, M1-A2, M1-A3, and M1-An increased to 8.8 MPa, 9.3 MPa, 9.1 MPa, and 9.6 MPa, representing improvements of 7.3%, 13.4%, 11.0%, and 17.1%, respectively. This increase in flexural strength is primarily because of the ICAs’ ability to chelate free Ca^2+^ and migrate them to the pores, where they react with SiO_3_^2−^ and other anions to form CaSiO_3_. These products fill the pores, densifying the mortar structure and enhancing flexural strength. Among the ICAs, An showed the highest Ca^2+^ chelation ability, with the fastest migration and reaction rate, resulting in the highest flexural strength for M1-An.

**Figure 13:**
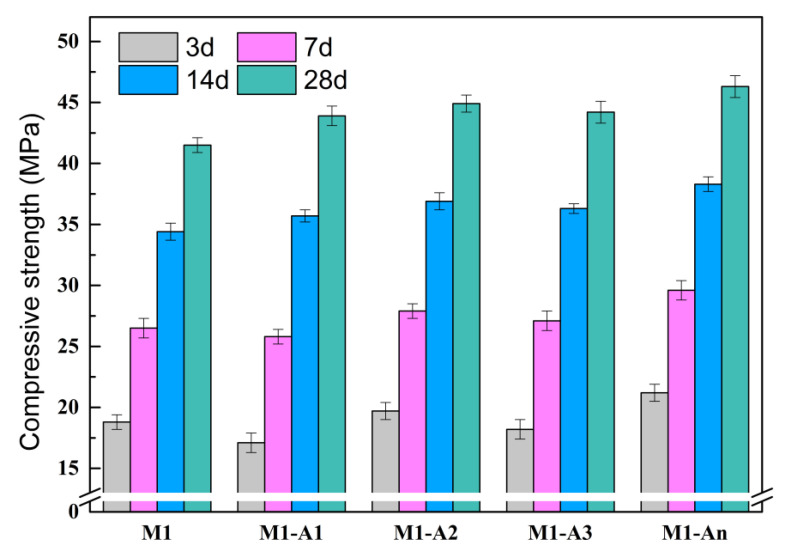
Compressive strength of mortars mixed with different ICAs.

The variations in compressive strength of mortars containing different ICAs during the course of the curing period are depicted in Figure 13. At 3 d of curing, the compressive strength of M1 was 18.8 MPa, while the compressive strengths of M1-A1, M1-A2, M1-A3, and M1-An were 17.1 MPa, 19.7 MPa, 18.2 MPa, and 21.2 MPa, respectively. This result suggests that A1 and A3, due to their retarding effects, reduced the hydration rate of cement, thereby slowing the early development of mortar compressive strength. In contrast, A2 and An promoted the formation of CaSiO_3_ during hydration, filling pores and forming a denser microstructure that increased the early compressive strength of the mortar. Over time, the inhibitory effects of A1 and A3 on cement hydration diminished, and after 14 d of curing, the hydration of C_3_S and C_2_S was nearly complete. This process generated a substantial amount of C-S-H gel, which filled the pores in the mortar, significantly enhancing density and compressive strength. The continued promotion of hydration by A2 and An further reduced harmful porosity and increased the strength of the mortar. Among the ICAs, An exhibited the strongest chelating and crystallization capacity, resulting in the highest compressive strength for M1-An.

#### 3.2.3. Chloride Ion Diffusion Coefficient

**Figure 14:**
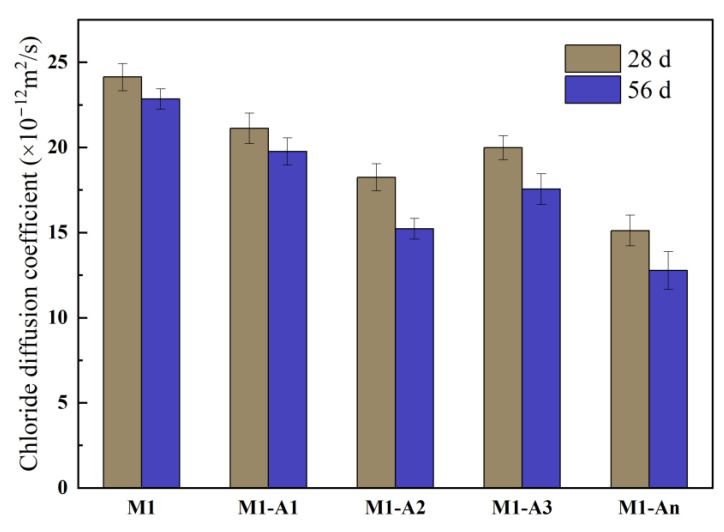
CIDCs of mortars mixed with different ICAs.

The CIDCs of mortars with different ICAs are shown in Figure 14. After 28 d of curing, the CIDC of M1 was 24.14 × 10^−12^ m^2^/s. Compared to M1, the CIDCs for M1-A1, M1-A2, M1-A3, and M1-An decreased by 12.5%, 24.4%, 17.2%, and 37.4%, respectively. This finding indicates that, during curing, ICAs can chelate with Ca^2+^ and react with anions within the pores and microcracks of the mortar to form crystalline products, such as CaSiO_3_, CaCO_3_, and AFt. These crystals fill cracks and pores, densifying the mortar structure and thereby enhancing its impermeability. Among the ICAs, An demonstrated the strongest ability to chelate Ca^2^⁺ and release it promptly during reactions with anions, leading to the formation of more products within the internal pores of M1-An, which significantly increased its impermeability. After 56 d of curing, the CIDCs of M1-A1, M1-A2, M1-A3, and M1-An were further reduced by 13.5%, 33.4%, 23.2%, and 44.1%, respectively, compared to M1. These results suggest that ICAs enable long-term chelation and crystallization reactions that continuously densify the mortar structure and improve its resistance to chloride ingress.

#### 3.2.4. Pore Structure

**Figure 15:**
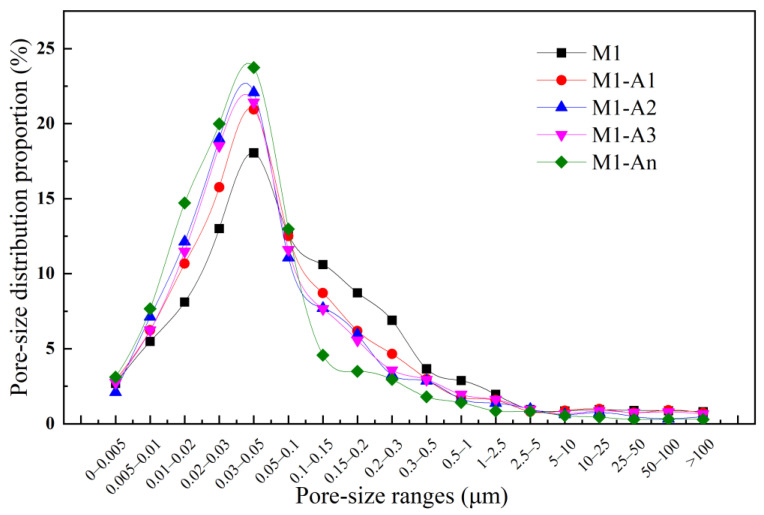
Pore size distribution of mortars mixed with different ICAs at 28 d.

Pores are categorized based on size into four types: harmless pores (<0.02 μm), less harmful pores (0.02–0.1 μm), harmful pores (0.1–0.2 μm), and highly harmful pores (>0.2 μm). The interconnectivity of harmful and highly harmful pore networks facilitates aggressive ion penetration while creating stress concentration sites, collectively degrading both mechanical performance and long-term durability [32,33]. Figure 15 shows the pore size distribution of mortars containing various ICAs after 28 d of curing. The inclusion of ICAs in the mortar increased the proportion of harmless and less harmful pores, with M1-An displaying the highest percentage of smaller pores. This effect was attributed to the ICAs’ ability to promote the formation of crystalline products, such as CaSiO_3_, CaCO_3_, and AFt, within the pores during curing. This process reduced the proportion of harmful pores, densifying the internal structure of the mortar. Additionally, An chelated more Ca^2+^, which then migrated to pores and formed crystalline precipitates, resulting in the densest internal structure for M1-An. For pores larger than 0.1 μm, the proportion in ordinary mortar gradually increased. This was primarily because the chelation and crystallization effects of the ICAs reduced harmful pore sizes, converting them into harmless pores, so the proportion of larger pores in ordinary mortar was higher than in mortars containing different ICAs.

**Figure 16:**
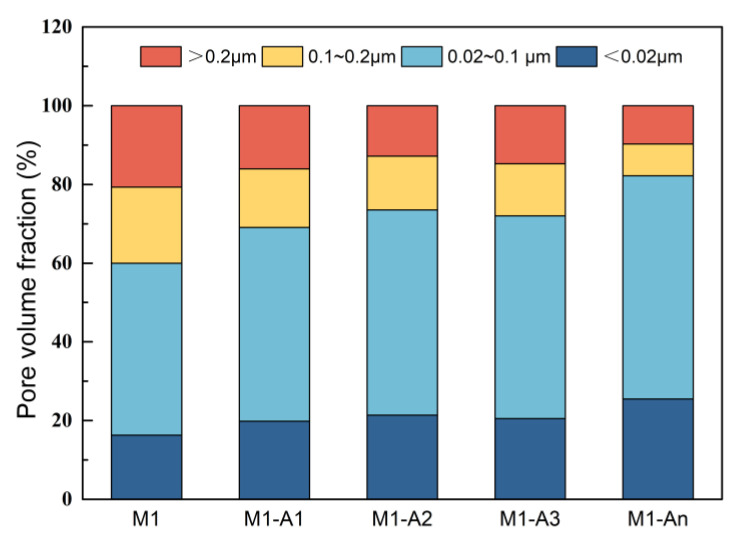
Pore volume fraction statistical results of mortars mixed with ICAs at 28 d.

Figure 16 presents the proportion of various pore types in mortars with different ICAs after curing for 28 d. The proportions of harmless pores in M1, M1-A1, M1-A2, M1-A3, and M1-An were 16.3%, 19.8%, 21.4%, 20.5%, and 25.5%, respectively. This result indicates that ICAs can reduce pore size by filling larger pores in the mortar. An, in particular, not only chelated Ca^2+^, facilitating the formation of CaSiO_3_ and CaCO_3_ crystals but also promoted cement hydration, resulting in the highest proportion of harmless pores in M1-An. Similarly, the proportions of highly harmful pores in M1, M1-A1, M1-A2, M1-A3, and M1-An were 20.7%, 16.0%, 12.8%, 14.8%, and 9.7%, respectively. These findings confirm that ICAs contribute to the conversion of harmful pores into harmless pores, thereby enhancing the mechanical strength and impermeability of mortar. Additionally, An was able to chelate multiple Ca^2+^ and migrate quickly, enabling it to rapidly promote the generation of crystallization products in macropores and reduce the proportion of highly harmful pores in the mortar.

#### 3.2.5. Internal Microscopic Morphology

**Figure 17:**
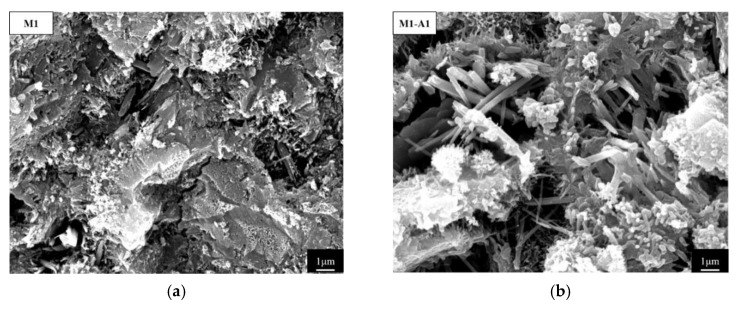

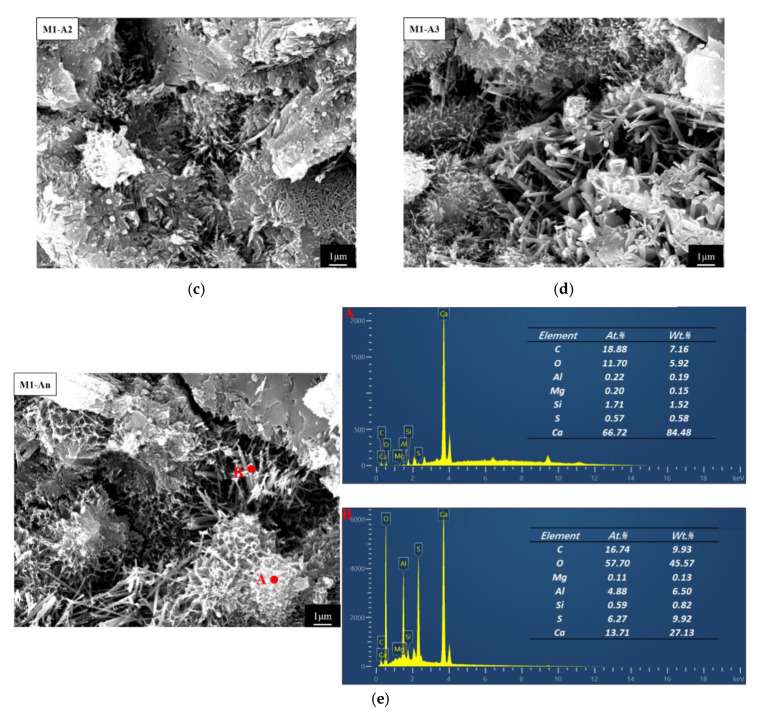
Internal microscopic morphology of mortar at 28 d: (**a**) M1; (**b**) M1-A1; (**c**) M1-A2; (**d**) M1-A3; and (**e**) M1-An. (A and B represent characteristic structural points).

**Figure 18:**
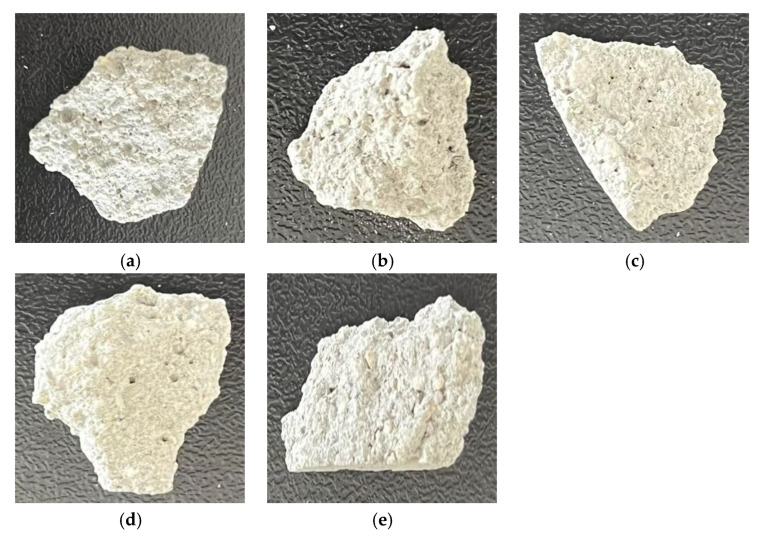
Sample images of mortar specimens for SEM imaging: (**a**) M1; (**b**) M1-A1; (**c**) M1-A2; (**d**) M1-A3; and (**e**) M1-An.

Figure 17 illustrates the internal microstructure of mortars with various ICAs at 28 d. M1 contained numerous pores with only minor flocculent products surrounding them. In contrast, the addition of ICAs improved the internal structure of the mortar. Although M1-A1 still exhibited some larger pores, needle-like products partially covered these pores, making the pores smaller. Similarly, M1-A2 and M1-A3 contained both flocculent and needle-like crystalline products that filled the pores, thereby enhancing the density of the mortar structure. Compared to the other mortars, M1-An demonstrated an abundance of crystalline products surrounding the pores, effectively reducing large pores. SEM-EDS analysis identified these products as primarily CaSiO_3_, CaCO_3_, and AFt. This densification was attributed to the ICAs chelating free Ca^2+^ in the matrix, which then migrates to the pores, where it not only reacts with cement hydration products to form CaSiO_3_ and AFt but also reacts with dissolved CO_2_ to produce CaCO_3_. This pore-filling action decreased the proportion of large pores in the mortar, enhancing both mechanical strength and impermeability of the mortars. The macroscopic photographs of mortar specimens prepared for SEM observation are presented in Figure 18.

## 4. Conclusions

In this paper, a high-efficiency ICA was synthesized, and the ion-chelating ability of different ICAs was investigated by measuring the conductivity of the respective solutions of this ICA and other ICAs (sodium gluconate, sodium maleate, and sodium citrate) under different pH, temperature, and ionic species conditions. The influences of different ICAs on the performance and microstructure of cement-based materials were evaluated through tests of hydration heat, mechanical strength, chloride ion diffusion coefficient, pore size distribution, and microstructural characterization. The main conclusions are as follows:

(1) The Ca^2+^ chelation capacity of the high-efficiency ICA was significantly enhanced compared to sodium gluconate, sodium maleate, and sodium citrate, with a chelation capacity of 813 mg/g. Under various temperature and pH conditions, the high-efficiency ICA effectively chelated Ca^2+^ and exhibited a chelation rate faster than the other three ICAs.
(2) The ICAs demonstrated varying chelation abilities for different metal ions, with the chelating ability for Ca^2+^ being the strongest and that for Al^3+^ being the weakest. The high-efficiency ICA possesses multiple chelation sites, resulting in superior chelation capacity for metal ions compared to other ICAs.
(3) Different ICAs had various impacts on the cement’s hydration process. Sodium gluconate and sodium citrate exhibited a retarding effect on cement, delaying the peak of the hydration heat. In contrast, sodium maleate and the high-efficiency ICA did not show a significant retarding effect, and the high-efficiency ICA facilitated the formation of C-S-H gel, resulting in the highest peak of hydration heat.
(4) The ICAs improved the mechanical strength and impermeability of the mortar, with the mortar containing the high-efficiency ICA exhibiting optimal performance. At 28 d, compared with ordinary mortar, the flexural strength and compressive strength of mortar mixed with high-efficiency ICA increased by 17.1% and 11.6%, respectively, and the chloride ion diffusion coefficient decreased by 37.4%.
(5) The ICAs effectively enhanced the pore size distribution and microstructure of the mortar. Compared to M1, the proportion of pores larger than 0.1 μm in M1-A1, M1-A2, M1-A3, and M1-An decreased by 22.7%, 33.8%, 30.1%, and 55.6%, respectively. SEM-EDS observations revealed that ordinary mortar contained larger and more loosely packed pores, while the mortar with ICAs exhibited a higher quantity of crystalline products within its pores. Notably, the high-efficiency ICA had a pronounced effect on optimizing the microstructure of the mortar, leading to the generation of a number of crystalline products, such as CaSiO_3_, within its pores.

## 5. Summary and Discussion of Results

This study investigated the effects of ion-chelating agents (ICAs) with varying chelation capacities on the properties and microstructure of cement-based materials. The results demonstrate that high-efficiency ICAs can significantly enhance the early hydration rate, mechanical strength, and impermeability of cement-based materials by forming crystalline products that fill pores and optimize the internal structure. These findings provide an effective solution for improving the durability and impermeability of concrete, particularly offering substantial application value for critical infrastructures such as bridges, tunnels, and high-rise buildings, which can consequently extend structural service life and reduce maintenance costs.

## Figures and Tables

**Table 1:** Chemical composition of cement (%).

	SiO_2_	Al_2_O_3_	CaO	MgO	SO_3_	Fe_2_O_3_	LOI
Cement	22.43	5.94	61.24	1.12	2.23	3.56	1.87

**Table 2:** Mix proportions of mortars by mass ratio.

	Cement	Water	Sand	A1	A2	A3	An
M1	100	40	200	0	0	0	0
M1-A1	100	40	200	0.1	0	0	0
M1-A2	100	40	200	0	0.1	0	0
M1-A3	100	40	200	0	0	0.1	0
M1-An	100	40	200	0	0	0	0.1

## Data Availability

The original contributions presented in this study are included in the article. Further inquiries can be directed to the corresponding author.
